# 3D convolutional neural networks-based segmentation to acquire quantitative criteria of the nucleus during mouse embryogenesis

**DOI:** 10.1038/s41540-020-00152-8

**Published:** 2020-10-20

**Authors:** Yuta Tokuoka, Takahiro G. Yamada, Daisuke Mashiko, Zenki Ikeda, Noriko F. Hiroi, Tetsuya J. Kobayashi, Kazuo Yamagata, Akira Funahashi

**Affiliations:** 1 Department of Biosciences and Informatics, Keio University, Kanagawa, 223-8522 Japan; 2 Faculty of Biology-Oriented Science and Technology, Kindai University, Wakayama, 649-6493 Japan; 3 Faculty of Pharmaceutical Sciences, Sanyo-Onoda City University, Yamaguchi, 756-0884 Japan; 4 Institute of Industrial Science, The University of Tokyo, Tokyo, 153-8505 Japan

**Keywords:** Software, Developmental biology

## Abstract

During embryogenesis, cells repeatedly divide and dynamically change their positions in three-dimensional (3D) space. A robust and accurate algorithm to acquire the 3D positions of the cells would help to reveal the mechanisms of embryogenesis. To acquire quantitative criteria of embryogenesis from time-series 3D microscopic images, image processing algorithms such as segmentation have been applied. Because the cells in embryos are considerably crowded, an algorithm to segment individual cells in detail and accurately is needed. To quantify the nuclear region of every cell from a time-series 3D fluorescence microscopic image of living cells, we developed QCANet, a convolutional neural network-based segmentation algorithm for 3D fluorescence bioimages. We demonstrated that QCANet outperformed 3D Mask R-CNN, which is currently considered as the best algorithm of instance segmentation. We showed that QCANet can be applied not only to developing mouse embryos but also to developing embryos of two other model species. Using QCANet, we were able to extract several quantitative criteria of embryogenesis from 11 early mouse embryos. We showed that the extracted criteria could be used to evaluate the differences between individual embryos. This study contributes to the development of fundamental approaches for assessing embryogenesis on the basis of extracted quantitative criteria.

## Introduction

During embryogenesis, cells repeatedly divide and dynamically change their positions in three-dimensional (3D) space^1^. In early embryos, cells are loosely connected to each other. At the 8-cell stage, the embryo becomes compact, and the cells form a spherical mass called a morula. The space inside the embryo spreads, and the morula becomes a blastocyst. Thus, embryo development is highly dynamic.

A robust and accurate algorithm to acquire the 3D positions of the cells with high temporal resolution would undoubtedly help to reveal the mechanisms of embryogenesis. The improved technologies for live-cell imaging enable obtaining high-quality and high-throughput time-series 3D fluorescence microscopic images^2–14^. In embryology, a number of studies have tried to acquire quantitative criteria such as chromosome numbers, the synchrony of cell division and the rate of development^15–17^. To analyse the time-series 3D microscopic images of developing embryos with fluorescently labelled nuclei, these studies used image segmentation. Segmentation algorithms in bioimage processing (such as filtering, thresholding, morphological operations, watershed transformation and mask processing^7,17–21^) require some parameter values. Because these algorithms are based on heuristic image-processing algorithms, they fail to detect an object in an image when this object does not fit the pattern that the algorithm can process. Even though the optimal parameter values depend on the features of each image and the microscopy system, these values are arbitrarily set by the analyst, and further optimisation tends to be neglected. As a result, it is hard to accurately acquire quantitative criteria with the existing heuristic image processing-based segmentation algorithms.

Various heuristic image processing algorithms have been used to investigate embryonic development^7,17–21^. For time-lapse observation of early-stage *D*rosophila embryos, Keller et al.^7^ implemented digital scanned laser light-sheet fluorescence microscopy in combination with incoherent structured-illumination microscopy (DSLM-SI) and performed nuclear segmentation of time-series images acquired by DSLM-SI. The algorithm was based mainly on heuristic image processing; the images had a high signal-to-noise ratio. *Drosophila* embryos are easily amenable to imaging because they are more transparent than the embryos of other model organisms, such as mice. Although Keller et al. were able to perform segmentation of time-series images, some limitations remained in their image processing. The segmentation accuracy dramatically decreased with the progress of embryo development: it was 95% for 2–4.5 h post-fertilisation (h.p.f.), 73% for 4.5–7 h.p.f. and 54% for 7–11.5 h.p.f. Analysis of time-series 3D fluorescence microscopic images is difficult: (i) fluorescence intensity decreases along the *z*-axis because the inner part of the embryo is not completely transparent; (ii) fluorescence intensity decreases with time because of fluorophore fading; and (iii) high spatial resolution cannot be achieved because a balance between cytotoxicity and the speed of photography needs to be maintained. The current low segmentation accuracy can be attributed to the fact that the variation in the spatiotemporal features of time-series 3D fluorescence microscopic images is not correctly grasped.

Deep learning algorithms named Convolutional Neural Networks (CNNs) may ameliorate these problems^22–32^. In general image processing, CNNs perform better than other algorithms^33^; a critical advantage of CNNs is automatic extraction of image features. CNNs have also been applied to bioimage segmentation algorithms, and the performance of CNNs was superior to that of the previous heuristic algorithms^22–25,28,32^. Ciçek et al.^23^ implemented 3D U-Net based on CNN and used it for segmentation of microscopic images of *X*enopus kidney tissue. The authors produced training data by manually annotating each image voxel-wise with “kidney tubule”, “inside kidney tubule”, or “background”. As a result of learning the training data, a high value of Intersection over Union (IoU), an evaluation metric for segmentation, was achieved (0.723). Ho et al.^28^ developed a CNN algorithm and used it to perform segmentation of 3D fluorescence microscopic images of labelled nuclei of rat kidney^28^; this algorithm achieved a voxel accuracy of 0.922. However, in both algorithms, some segmented nuclear regions are fused with other regions, which disturbs the acquisition of quantitative criteria from bioimages.

**Fig. 1:**
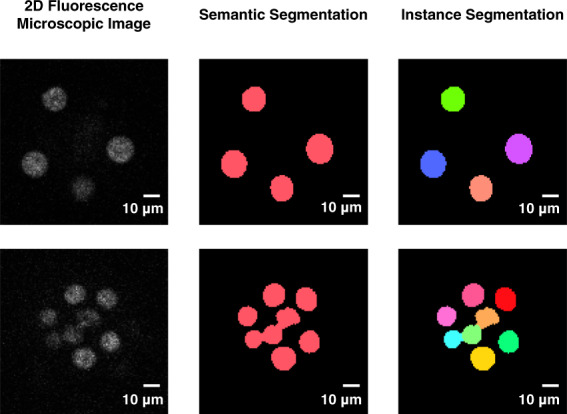
Conceptual diagram of different segmentation algorithms. In a 2D fluorescence microscopic image, all objects to be segmented are of the same class (nucleus). Semantic segmentation assigns the same label to all objects of the same class, whereas instance segmentation assigns different labels. When the objects are sufficiently separated in space (upper panels), segmentation of both types is accurate. When objects are adjacent or overlap (lower panels), semantic segmentation fuses the object regions, but instance segmentation does not.

The segmentation algorithms just mentioned are based on Fully Convolutional Networks (FCNs), which consist only of convolutional layers in CNNs, and the segmentation methodology of FCNs is called semantic segmentation^34^. Because semantic segmentation assigns the same label to the objects of the same class (Fig. 1), the regions are fused when neighbouring or overlapping objects are segmented^35,36^. Therefore, semantic segmentation is appropriate for the tissue, but not for individual cells or organelles. In this study, we focused on the other segmentation methodology, called instance segmentation^27,31,37–39^, which adds a different label to each object of the same class (Fig. 1) and is suitable for segmentation of cells and nuclei. This property of instance segmentation avoids the fusion of cells and is especially important for the analysis of stages such as morula or blastocyst, in which the cells are located close to each other; instance segmentation makes it possible to accurately acquire quantitative criteria of embryogenesis.

Here, we developed Quantitative Criteria Acquisition Network (QCANet), a new CNN-based instance segmentation algorithm for 3D fluorescence microscopic images of cell nuclei of early embryos. Its simple structure combines conventional semantic segmentation algorithms and it can be easily applied to bioimage analysis. We prepared a dataset that sampled early development of 11 mouse embryos with nuclei fluorescently labelled with mRFP1 fused to the chromatin marker histone H2B^2^ and trained QCANet to perform instance segmentation of 3D fluorescence microscopic images from this dataset. A comparison of the accuracy of the trained models using four other mouse embryos as a test dataset showed that QCANet was superior to 3D Mask R-CNN^39^, which is the state-of-the-art of the instance segmentation algorithm, in terms of segmentation accuracy called IoU, SEG and MUCov. To check whether QCANet performs segmentation with high accuracy in other species, we used the datasets of developing embryos of *Caenorhabditis elegans* and *D*rosophila melanogaster. QCANet showed high segmentation accuracy on almost all metrics. Using trained QCANet, we extracted quantitative criteria of mouse development based on the accurately acquired shapes of cell nuclei without fusion and quantitatively evaluated the differences between individual embryos. We also classified each cell nucleus segmented by QCANet as belonging to an inner cell or outer cell and demonstrated that the estimated ratio of the numbers of inner and outer cells can serve as a proxy of differentiated cells in morulae and blastocysts.

## Results

### Evaluation of 3D instance segmentation

**Fig. 2:**
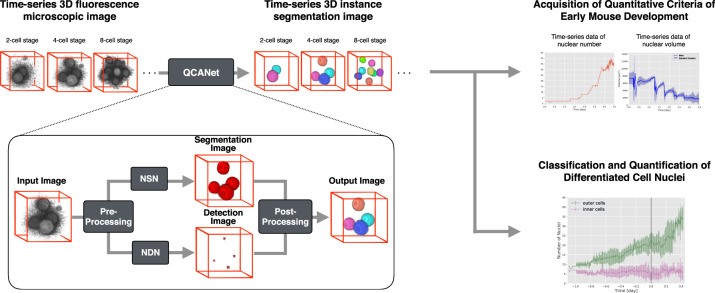
Flow diagram of the QCANet algorithm and downstream analysis of quantitative criteria acquisition from QCANet segmentation results. QCANet performs instance segmentation from time-series 3D fluorescence microscopic images of early-stage embryos as an input. QCANet first pre-processes the input image at each time point. The pre-processed image is then processed in parallel by the Nuclear Segmentation Network (NSN), which segments the nuclei, and the Nuclear Detection Network (NDN), which identifies them. The nuclear region segmented by NSN is divided by marker-based watershed in post-processing using the nuclear centre identified by NDN. By performing instance segmentation at each time point, QCANet acquires a time-series instance segmentation image. We used time-series segmentation images acquired by QCANet to acquire quantitative criteria of early mouse development. QCANet can also be used for classification and quantification of differentiated cell nuclei.

The implemented algorithm QCANet is a tool for instance segmentation of 3D fluorescence microscopic images (Fig. 2). QCANet consists of two subnetworks: Nuclear Segmentation Network (NSN) and Nuclear Detection Network (NDN). Because instance segmentation in QCANet relies on nuclear detection by NDN, we compared the segmentation accuracy of QCANet with that of QCANet without (w/o) NDN. We also compared QCANet with the conventional 3D segmentation algorithms 3D U-Net^23^ and 3D Mask R-CNN^39^. The latter is a 3D extension of the state-to-the-art instance segmentation algorithm Mask R-CNN^37^. These algorithms learned the same dataset, which included 11 early-stage mouse embryos. We used 11-fold cross-validation with 121 samples of 3D fluorescence microscopic images (Supplementary Fig. 1); 10 embryos (110 samples) were used as training data and 1 embryo (11 samples) as validation data (Supplementary Fig. 2, embryo split). In addition, we prepared a test dataset of four early mouse embryos (44 samples) with a different observation environment from that of the training and validation datasets (Supplementary Tables 1 and 2). The trained model with the highest IoU in cross-validation was used to analyse the test dataset, and we evaluated its segmentation accuracy.

IoU is conventionally used to evaluate segmentation accuracy because it comprehensively measures false-positive and false-negative rates^23^. However, because IoU is calculated for each image, it cannot evaluate whether or not segmentation is accurate (i.e., nuclei are not fused) and is thus unsuitable for evaluating instance segmentation. A metric called SEG^40^ represents the average of the IoU of each instance by the sum of the numbers of correct nuclear regions. Another metric, called Mean Unweighted Coverage (MUCov)^41^, can evaluate individual segmented nuclear regions and represents the average of the IoU of each instance by the sum of the numbers of segmentation regions. SEG is used to evaluate the absence of false-negative instances of segmentation, whereas MUCov is used to evaluate the absence of false-positive instances.

**Fig. 3:**
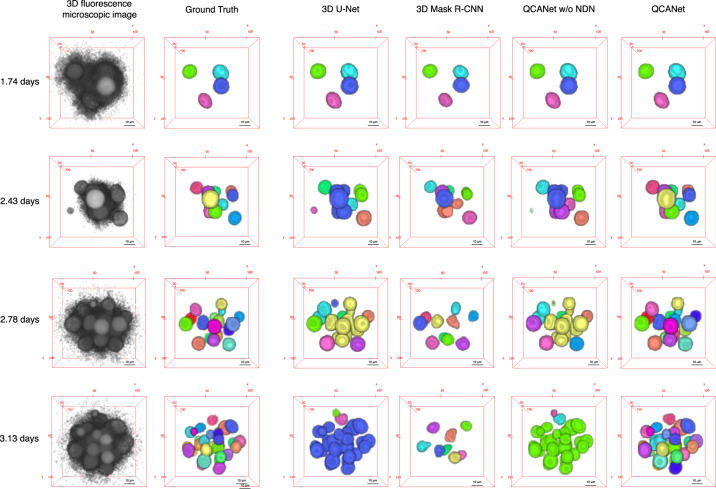
Qualitative comparison of segmentation for mouse embryo by 3D U-Net^23^, 3D Mask R-CNN^39^, QCANet without (w/o) NDN and QCANet. Segmentation of 3D fluorescence microscopic images of mouse embryo at four stages is shown. Each colour represents an individual segmented nuclear region. Days elapsed after the pronuclear stage are indicated.

In 11-fold cross-validation, the values of IoU, SEG and MUCov of QCANet exceeded those of the other algorithms (Supplementary Table 3). Each value does not deviate largely among embryos. We also analysed the test dataset with the model showing the highest IoU for the validation dataset, and QCANet outperformed the other algorithms on all metrics (Table 1). Visualisation of the segmentation results showed that QCANet detected nuclei accurately, whereas 3D U-Net and QCANet w/o NDN fused some nuclei to each other and 3D Mask R-CNN missed several nuclei (Fig. 3). We concluded that QCANet has a small false-negative error in nucleus detection and allows accurate segmentation to acquire the quantitative criteria of early mouse development.

**Table 1:** Quantification of the segmentation accuracy of QCANet, QCANet without (w/o) NDN, 3D U-Net^23^ and 3D Mask R-CNN^39^ using IoU (metric for semantic segmentation), and SEG and MUCov (metrics for instance segmentation).

Algorithm	IoU	SEG	MUCov
3D U-Net	0.702 (0.054)	0.206 (0.161)	0.243 (0.170)
3D Mask R-CNN	0.558 (0.195)	0.476 (0.248)	0.607 (0.144)
QCANet w/o NDN	0.742 (0.060)	0.533 (0.265)	0.520 (0.173)
QCANet	0.746 (0.060)	0.710 (0.109)	0.721 (0.085)

Each value (mean and standard deviation) was calculated on the basis of the test dataset analysis.

To evaluate the temporal robustness of the accuracy of instance segmentation by QCANet, we evaluated the training of QCANet by 11-fold cross-validation with the dataset divided into time points (Supplementary Fig. 2 time split). The average values were 0.808 for IoU, 0.761 for SEG and 0.787 for MUCov (Supplementary Fig. 3), almost the same as in Supplementary Table 1. Thus, we demonstrated the temporal robustness of the accuracy of instance segmentation by QCANet during embryo development.

Using QCANet, we performed instance segmentation of time-series 3D fluorescence microscopic images of 11 mouse embryos (Supplementary Video 1) and qualitatively showed that QCANet correctly determined the nuclear regions and accurately performed instance segmentation without fusion of cell nuclei. Although developing mouse embryos have complex characteristics such as the rate of development, nucleus arrangement, cell and nucleus shape, and fluorescence intensity, the QCANet performance was robust.

### Applicability of QCANet to developing embryos of *C. elegans* and *D. melanogaster*

We tested whether QCANet could perform segmentation with high accuracy for other model species. We used public datasets of developing embryos of *C. elegans* and *D. melanogaster*^40,42–44^. The datasets of each species were split into the training (2 embryos), validation (1 embryo) and test (1 embryo) data. Both datasets consisted of time-series 3D fluorescence images acquired by live-cell imaging during development.

**Fig. 4:**
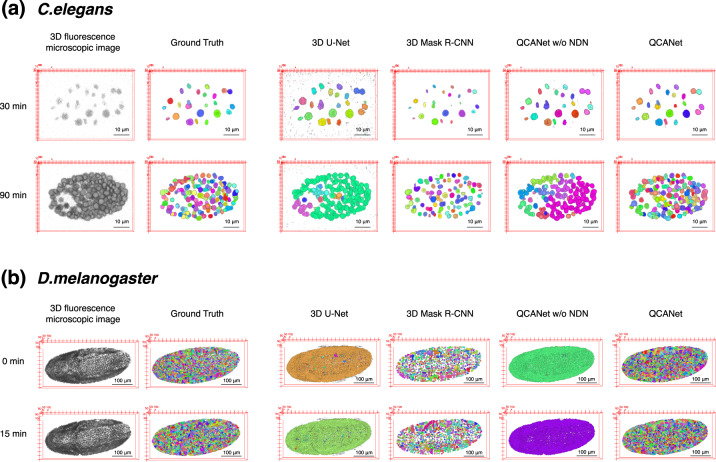
Qualitative comparison of segmentation for *C. elegans* and *D. melanogaster* embryos by 3D U-Net^23^, 3D Mask R-CNN^39^, QCANet without (w/o) NDN and QCANet. Each colour represents an individual segmented nuclear region.

The *C. elegans* dataset contained images acquired from the two-cell stage to a stage including ~300 cells or more. We manually created a ground truth of segmentation by sampling 5 or 7 time points per embryo. This ground truth was used for training and evaluation. In the training step, we performed 3-fold cross-validation (Supplementary Table 4) and selected the model with the highest IoU (the metric for the accuracy of semantic segmentation) to perform segmentation for the test embryo. Although the value of IoU was the highest with 3D U-Net, the values of SEG and MUCov (metrics for the accuracy of instance segmentation) were the highest with QCANet and 3D Mask R-CNN, respectively (Table 2). A comparison of segmentation by different algorithms is shown in Fig. 4a. In 3D U-Net, a large fraction of nuclei was fully fused; such fusion prevents acquisition of quantitative criteria, therefore, 3D U-Net is not applicable to the analysis of developing embryos. In 3D Mask R-CNN, nuclei were not fused, but many of them were missed; this fact was also supported by the low SEG value. Missing nuclei lead to the incorrect estimate of the cell number. In QCANet, nuclei were not fused or missing; thus, QCANet is the most accurate among the tested algorithms in acquiring quantitative criteria.

**Table 2:** Quantification of the segmentation accuracy of QCANet, QCANet without (w/o) NDN, 3D U-Net^23^ and 3D Mask R-CNN^39^ using IoU (metric for semantic segmentation), and SEG and MUCov (metrics for instance segmentation) for *C. elegans* and *D. melanogaster* embryos.

	*C.elegans*			*D.melanogaster*		
Algorithm	IoU	SEG	MUCov	IoU	SEG	MUCov
3D U-Net	0.549 (0.226)	0.290 (0.205)	0.002 (0.001)	0.637 (0.016)	0.000 (0.000)	0.004 (0.001)
3D Mask R-CNN	0.476 (0.075)	0.290 (0.155)	0.355 (0.145)	0.344 (0.006)	0.085 (0.007)	0.218 (0.010)
QCANet w/o NDN	0.500 (0.179)	0.257 (0.141)	0.124 (0.079)	0.514 (0.024)	0.002 (0.000)	0.012 (0.003)
QCANet	0.508 (0.160)	0.386 (0.069)	0.340 (0.171)	0.516 (0.024)	0.243 (0.010)	0.260 (0.010)

Each value (mean and standard deviation) was calculated on the basis of test dataset analysis.

The *D. melanogaster* dataset contained images acquired from a stage with ~2500–3000 cells or more. We manually created a ground truth of segmentation by sampling 2 time points per embryo. The ground truth was used for training and evaluation. In the training step, we performed 3-fold cross-validation (Supplementary Table 5). Then, we selected the model with the highest IoU to perform segmentation for the test embryo. The highest IoU was obtained with 3D U-Net, and the highest SEG and MUCov with QCANet (Table 2). A comparison of segmentation by different algorithms is shown in Fig. 4b. As in *C. elegans*, QCANet did not fuse or miss nuclei. This result confirmed that QCANet robustly performs accurate instance segmentation even in embryos containing thousands of cells (Fig. 4b).

Overall, these results indicate that QCANet is superior to all of the other algorithms in terms of performing instance segmentation of cell nuclei of various developing embryos.

### Acquisition of quantitative criteria of early mouse development

**Fig. 5:**
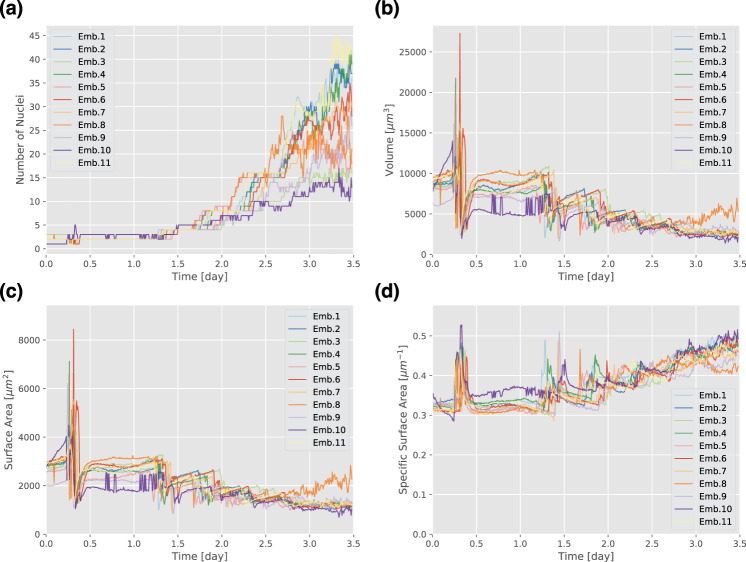
Quantitative criteria of mouse development extracted by QCANet from time-series data. **a** Nuclear number. **b** Nuclear volume. The tendency of nuclear volume to rapidly decrease and then recover may indicate mitosis. **c** Nuclear surface area. This time course also captures the features of mitosis. **d** Nuclear specific surface area. The tendency of nuclear specific surface area is rapidly increasing immediately before mitosis and then recover after division.

Using these time-series instance segmentation images by QCANet, we first extracted the time-series data of the nuclear number, volume, surface area and specific surface area (Fig. 5). We found a periodical tendency of sharp decreases in nuclear volume followed by its partial recovery. This tendency was consistent with the increase in the number of cells nuclei (Fig. 5a, b and Supplementary Fig. 4). We concluded that QCANet extracts feature characteristic of mitosis. The nuclear volume from the pronuclear to 2-cell stage (0–1.3 days) was 5000-10,000 μm^3^ (Fig. 5b). The volume of the mouse embryo at the 2-cell stage is ~56,000 μm^3^ ^45^; thus, our estimate of the nuclear volume appeared to be reasonable. The nuclear surface area followed a similar tendency, probably because the nucleus is spherical (Fig. 5c), whereas the tendency of the nuclear specific surface area was opposite (Fig. 5d). Because the specific surface area increases as the sphere volume decrease, this result showed that the cell nuclei were becoming smaller yet maintained their spherical shape during development. The similar tendency was confirmed by the previous report^46^. The specific surface area increased because the shape of the nuclear region changed rapidly at the beginning of mitosis (Supplementary Fig. 5); also, the volume decreased dramatically and then partially recovered after mitosis.

**Fig. 6:**
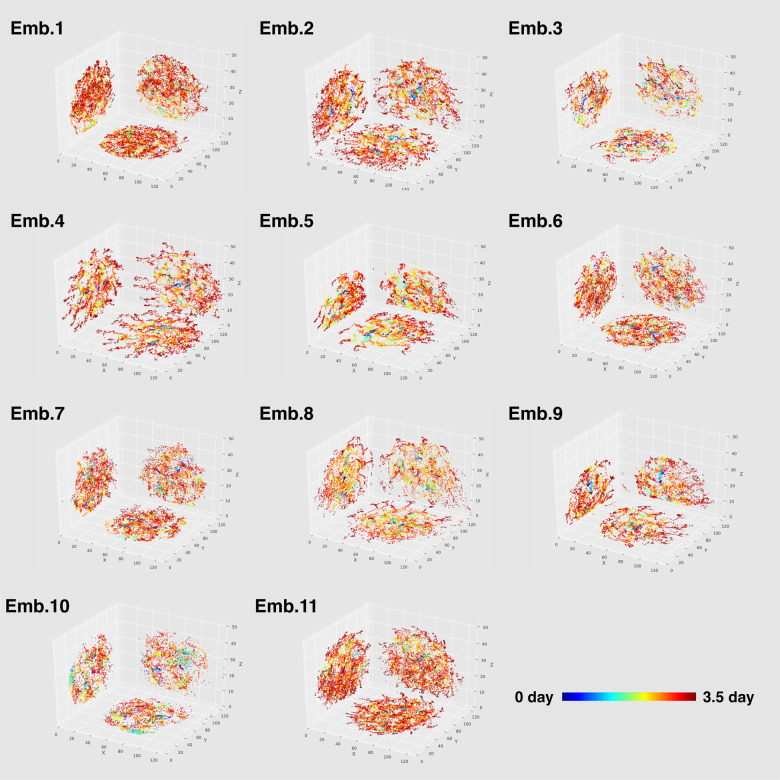
The time-series data of the nuclear centre of gravity coordinates extracted by QCANet for 11 mouse embryos. Colour shift from cold to warm indicates the course of development. Over time, the internal clearance widens, indicating blastocyst formation. In each panel, the results are displayed in 2D (XY, XZ and YZ cross-sections).

Second, we extracted the time-series data of the nuclear centre of gravity coordinates (Fig. 6). During the development from morula to blastocyst, the internal space expands and cells of the outer layer of the blastocyst (trophectoderm^47^) become the source of extraembryonic tissue. We observed an expansion of the internal space during blastocyst formation. We calculated the space fill factors from all-time data of the nuclear centre of gravity coordinates in each embryo (Supplementary Fig. 6); the values of these factors indicate the position bias of cell nuclei in the developing embryo. Many space fill factors reached maximum near the embryo centre, indicating the persistence of cell nuclei there during the 2–4-cell stages.

**Fig. 7:**
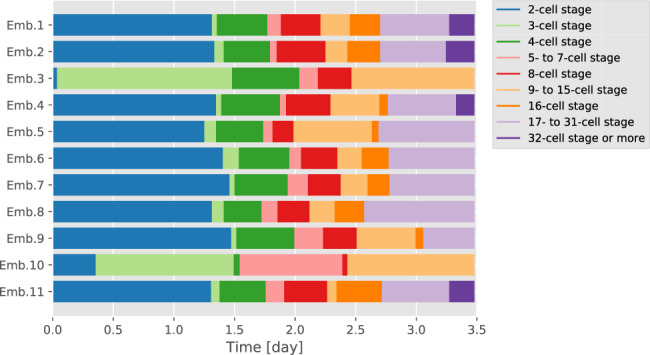
Synchrony of cell division. Each colour represents an embryo stage. If cell division is synchronous, the 2-, 4-, 8-, 16- and 32-cell stages are longer than the other stages. In Emb.3 and Emb.10, cell divisions were not synchronous.

Third, we extracted the synchrony of cell division (Fig. 7). An embryo at the 32-cell stage or more at 3.5 days is a normal embryo at the blastocyst stage^48,49^. Embryos 3 and 10 did not reach the 16-cell stage. The interstage duration (3-, 5- to 7-, 9- to 15-, 17- to 31-cell stage) in these embryos tended to be longer than in the others. Therefore, cell division in embryos 3 and 10 was not synchronised.

Thus, we showed that we can extract quantitative criteria of early mouse development using QCANet and quantitatively evaluate differences between individual embryos.

### Classification and quantification of differentiated cell nuclei

The first cell differentiation in early mouse development begins with the separation of the inner cell mass (ICM), which will form the embryo body, and trophectoderm (TE), which will form the placenta. This differentiation begins at the morula stage^50^. The cell fate choice between the ICM or TE is correlated with the spatial arrangement of cells inside or outside each region after the morula stage^51,52^. However, there are no reports on the temporal changes in the ratio of outer cells to inner cells within each stage, the morula and the blastocyst. Therefore, we quantified the temporal changes in the numbers of the inner and outer cells from the 16-cell stage, which we considered as morula, to the blastocyst.

**Fig. 8:**
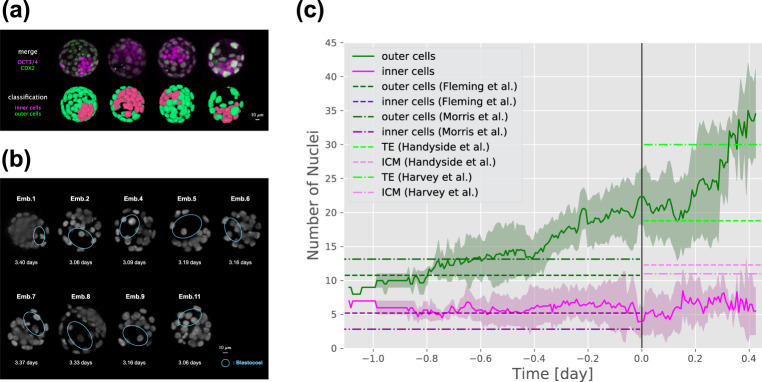
Classification of cells during the morula and blastocyst stages into inner cells and outer cells. **a** The results of sorting of the blastocyst nuclei into those of the inner and outer cells in comparison with the results of immunofluorescent staining of OCT3/4 and CDX2 in the same embryos. Oct3/4 is expressed in ICM (magenta) and Cdx2 in TE (green). **b** Time to reach the blastocyst stage for each embryo. Blue circles show the blastocoel, whose formation indicates the blastocyst stage. **c** Time-series data of nuclear numbers. The origin of the time axis represents the time at which the blastocysts formed. Lines represent the mean and the lighter shaded areas represent standard deviation. Horizontal dotted lines represent data from previous studies^47,51,53,54^.

Differentiated cells in the blastocyst can be used to reliably distinguish between the inner and outer cells^52^. To establish a boundary between the inner and outer cells, we used immunofluorescence staining of four blastocysts with antibodies against OCT3/4 and CDX2, transcription factors specifically expressed in the ICM and TE, respectively. Then, using the centre of gravity coordinates of the nuclei determined with the H2B probe, we determined a spherical boundary around the centre of the embryo that separates the inner and outer regions. Using the determined boundary, we classified the nuclei in time-series images into those belonging to the inner cells or outer cells from the 16-cell stage to the blastocyst stage (Supplementary Fig. 7a). The inner and outer cell areas classified at a ratio of 0.4 coincided with the experimentally confirmed ICM and TE areas (Fig. 8a and Supplementary Fig. 7b).

Nine embryos (except embryos 3 and 10) had normal cleavage synchronisation and reached the blastocyst stage (Fig. 7). The time at which these embryos formed the blastocoel was defined as the time at which they reached the blastocyst stage (Fig. 8b); the inner cells/outer cells were classified at this time, which was defined as 0 days. Then, the inner cells/outer cells were classified at time points before and after 0 days, and the temporal changes in the number of nuclei were extracted (Fig. 8c). After 0 days, the number of nuclei belonging to outer cells, but not to inner cells, increased rapidly with time. Quantification by immunofluorescence staining of ICM/TE cells in the early blastocyst^53,54^ did not allow the acquisition of live cells in time series, i.e., it is not available to count the ICM/TE cell number at various time points. Previous studies have quantified the inner and outer cells in the morula^47,51^; however, as with the blastocyst, the time variation is unknown. Before 0 days (at the morula stage), the number of the nuclei of outer cells increased gradually with time and that of inner cells remained constant.

## Discussion

Segmentation is an important and challenging task of bioimage analysis aimed at uncovering biological phenomena such as embryogenesis. QCANet was able to solve the problem of nucleus fusion in the test data, which was not solved by 3D U-Net, and that of missed nucleus detection, which was not solved by 3D Mask R-CNN. The test data were different from training and validation data in terms of imaging conditions. Thus, QCANet can robustly perform instance segmentation for images acquired under different imaging conditions. QCANet performed instance segmentation with higher accuracy than the other algorithms in three model organisms, mouse, *C. elegans* and *D*. melanogaster. Thus, QCANet is the best algorithm in terms of segmenting cell nuclei during embryonic development in different species.

QCANet can qualitatively classify development into normal versus abnormal using quantitative criteria extracted from early mouse embryos. Embryos reaching the blastocyst stage (32 cells or more) are considered to be normal^48,49^. Embryos 3 and 10 reached only the 9- to 15-cell stage, whereas other embryos had already passed the 16-cell stage (Fig. 7). The duration of the 9- to 15-cell stage in embryos 3 and 10 was much longer than in the other embryos. These results indicate that embryos 3 and 10 lost the ability to proceed to the next developmental stage with normal rate. In these embryos, the developmental abnormality started early, because the 3- and 5- to 7-cell stages were already much longer than in the other embryos. The fate of the mouse embryo, in particular whether it reaches the blastocyst stage, is greatly affected by the initial cell division pattern^1,55^; synchronicity of the 2nd and 3rd mitoses within 5.8 h has been proposed as one of the criteria for classifying normal human embryos^56^. In normal embryos, the second and third cell synchrony is good, that is, the duration of the 3-cell stage is short. The duration of the 3-cell stage exceeded 1 day in embryos 3 and 10 but was within 5.8 h in the other embryos (Fig. 7). In embryo 10, the specific surface area from 0.5 to 1.5 days was larger than in the other embryos (Fig. 5d), and the values and fluctuations of the space fill factors were smaller (Supplementary Fig. 6). Two criteria, the number of cells and duration of each stage, could be used to qualitatively classify embryogenesis into normal or abnormal.

Comparison of the accuracy of the extraction of the synchrony of cell divisions showed that QCANet w/o NDN detected more embryos with long interstage duration (Supplementary Fig. 8a) than QCANet did (Fig. 7). In embryo 4 at 0.08 days, QCANet w/o NDN showed an apparent false-positive nucleus (three nuclei in total), whereas QCANet recognised it as a 2-cell-stage embryo (Supplementary Fig. 8b). In embryo 9 at 0.08 days, QCANet w/o NDN extracted a false-positive nucleus and defined embryo 9 as the 4-cell stage (Supplementary Fig. 8b), whereas QCANet recognised 3 cells (Fig. 7) and the absence of the synchrony of cell division. Recognition of false-positive nuclei is the major barrier to accurate extraction of the synchrony of cell divisions by QCANet w/o NDN. QCANet overcomes this barrier and accurately extracts the synchrony of cell divisions, an important criterion in embryology.

The values of the quantitative criteria acquired by QCANet varied among embryo. There were two possible causes for this variation: biological variability and segmentation errors made by QCANet. To examine whether this variability was caused by biological variability, we created the correct answers for the number of cell nuclei, volume, surface area and specific surface area at 11 time points based on the ground truth. We found that the number of cell nuclei varied among embryos from 1.4 days after fertilisation, and other criteria varied among embryos at all time points (Supplementary Fig. 9, cross mark). Then, we created the correct answers for the centre of gravity coordinates of cell nuclei and the synchrony of cell division, and examined the variability among embryos. We found that the values of the centre of gravity coordinates (Supplementary Fig. 10a) and the synchrony of cell division (Supplementary Fig. 11) varied in each embryo. Therefore, the quantitative criteria had biological variability among early mouse embryos.

To examine whether QCANet accurately captured this biological variability, we tested its segmentation error. The segmentation accuracy of QCANet was considerably lower in the early (~0.35 days) and late (after ~2.8 days) stages than in the other stages (Supplementary Fig. 12a). The decrease in accuracy at ~0.35 days was because one of the four embryos in the test data had low accuracy of segmentation (IoU, SEG and MUCov values were 0.418); in this embryo, the fusion of the male and female pronuclei occurred at this time point (Supplementary Fig. 12b). After ~2.8 days, the accuracies for all four embryos of the test data were consistently decreasing. For the number of cell nuclei, volume, surface area and specific surface area, the comparison of QCANet values with the correct answers showed that the effect of QCANet error was small before 2.8 days, except for ~0.35 days (Supplementary Fig. 9). For the centre of gravity coordinates, we qualitatively confirmed that the difference between correct values and values extracted by QCANet was almost consistent (Supplementary Fig. 10). Many fewer gravity coordinates of nuclei were extracted by QCANet than in the ground truth (e.g., in Emb. 8 in Supplementary Fig. 10, the number of red dots was considerably lower in the QCANet results than in the ground truth). This trend was caused by the high false-negative error of QCANet at the late stages of development. For the synchrony of cell division, the cell stage determined by QCANet was consistently lower than that of the ground truth from 2.8 days for all embryos (Supplementary Fig. 11). This trend could be caused by nuclei missing by QCANet. Overall, we concluded that the quantitative criteria acquired by QCANet accurately captured biological variability except at ~0.35 days and after ~2.8 days.

The number of the nuclei of outer cells specifically increased and that of inner cells almost remained constant from the morula to the blastocyst stage. This could be induced by a difference in the manner of cell division in which outer cells may divide only into outer cells, whereas inner cells may divide into inner cells and outer cells. TE proliferates by repeated fission at the morula and blastocyst stages^52,57^. ICM but not TE has the ability to divide into ICM and TE^58^. We discovered a continuous increase in the number of outer cells, which might be required for proper development.

In this study, we attempted to count the cell number in ICM and TE by using a single probe, H2B. The number of inner and outer cells was consistent with that in the ICM and TE regions determined with specific markers. These results suggest that correct classification of ICM and TE can be achieved by using the indices of area and cell number. Several probes have been used in previous studies to quantify ICM and TE^52,57^. Our results show that the H2B probe alone is sufficient not only to quantify cell nuclei (and therefore cell number) but also to classify ICM and TE.

Polar bodies have a nucleus but hardly any cytoplasm; they are formed during oocyte meiosis and slowly degenerate during embryo development and disappear naturally^59–61^. Polar bodies may not be related to normal development and should be excluded from segmentation targets. However, polar bodies tend to be extracted by image processing because they produce fluorescent protein encoded by the microinjected mRNA. In two cases, QCANet excluded the nuclei of polar bodies from segmentation: (i) both NSN and NDN excluded these nuclei (Supplementary Fig. 13a–c), and (ii) NSN identified the nuclei of a polar body, but NDN excluded them (Supplementary Fig. 13d–f). Why did QCANet exclude the polar body in the second case? NSN and NDN identified the nuclei independently. The watershed process was performed using the result of NDN to divide the nuclear region segmented by NSN. When NDN identified the false-positive error of NSN, the nucleus of the polar body was excluded in post-processing. This result shows that QCANet performs high-quality analysis of bioimages.

The role of polar bodies in the development has been discussed for a long time^59,60^, but there is no clear answer as to why they exist in the embryo^61^. Because QCANet recognised the nuclei of polar bodies, it seems possible to trace only polar bodies during development; thus, QCANet will be a powerful tool in developmental biology. Yet, how QCANet recognises the polar bodies was not evident because the regression of deep learning was too complicated. Some studies have tried to analyse learned features^62,63^. It was reported that each layer in the neural network has a role in image processing such as filtering^64^. The results of these studies suggest that the regression by deep learning could be replaced by a combination of different image processing approaches. If this combination is revealed and the layer that has a role in distinguishing nuclei of embryonic cells from those of polar bodies is determined, the mechanism of recognition of polar bodies will be uncovered.

Because QCANet is not an end-to-end learning algorithm, NSN and NDN need separate parameter tuning and training. Therefore, QCANet needs to be improved and further developed to become an end-to-end learning algorithm. 3D Mask R-CNN is a state-of-the-art in instance segmentation and is an end-to-end learning algorithm. On the other hand, QCANet is better than 3D Mask R-CNN for instance segmentation of developing embryos, especially at avoiding false-negative errors in nuclear detection during nuclear segmentation. The false-negative errors of 3D Mask R-CNN make it difficult to accurately quantify cell number-dependent events such as cell division during development. Therefore, QCANet rather than 3D Mask R-CNN is suitable for obtaining quantitative criteria of early mouse development.

We also compared QCANet and 3D Mask R-CNN from the viewpoint of ground truth production cost. The dataset used for QCANet training requires annotation of semantic segmentation of each nucleus and the nuclear centre region. On the other hand, the dataset used for 3D Mask R-CNN training requires annotation of instance segmentation of each nucleus and the bounding box of each instance. Compared with instance segmentation, semantic segmentation does not require the addition of precise per-instance boundary annotations and different labels. Therefore, the cost of creating the ground truth for QCANet training is lower than that for 3D Mask R-CNN.

Segmentation is an indispensable technology for the quantification of vital phenomena, but it does not reach the accuracy necessary for automation and its results need to be evaluated by biologists. It is more expensive to manually segment a region missed because of a false-negative error than to remove a region detected because of a false-positive error. Therefore, our study demonstrates the usefulness of QCANet, which has few false-negative errors.

Although this study focused on early mouse embryogenesis, we demonstrated that QCANet could accurately perform segmentation for cell nuclei of developing embryos of other species. The *C. elegans* and *D. melanogaster* datasets had a wide range of developmental stages from two to several hundred cells and several thousand cells. Thus, QCANet is applicable across a wide range of developmental stages and is a very useful foundational tool in embryology.

We expect that QCANet will considerably improve the quality and throughput of embryologic analysis. Two major future challenges have to be considered. (i) The number of cell nuclei occasionally decreases with time (Supplementary Fig. 9a and Fig. 8c), likely because of false-negative detection errors in QCANet. Indeed, the value of SEG in QCANet decreased after 2.8 days in mouse development (Supplementary Fig. 12a). Besides, the segmentation accuracy of QCANet was low when the nucleus shape dynamically changed, e.g., as a result of the fusion of the male and female pronuclei at ~0.35 days (Supplementary Fig. 12b). Therefore, future improvements to QCANet will be needed to reduce segmentation errors and false-negative detection errors in these cases. (ii) QCANet performs segmentation at each time point independently and does not perform tracking. Addition of a tracking algorithm to the segmentation algorithm of QCANet would allow applying QCANet to cell lineage analysis. We believe that incorporating a tracking algorithm into QCANet is an important challenge for the future.

## Methods

### Ethics Statement

Male and female ICR strain multiclonal hybrid mice (Jcl: MCH (ICR)) were used for gamete preparation for training dataset of 11 mouse embryos. Male and female ICR (slc: ICR) strain were used for gamete preparation for test dataset of 4 mouse embryos. All animal experiments were conducted according to the Guide for the Care and Use of Laboratory Animals and were approved by the Institutional Committees of Laboratory Animal Experimentation of Osaka University and Kindai University (permit number: KABT-31-016).

### Animals

ICR mice (12–16 weeks old) were obtained from Japan SLC, Inc. (Shizuoka, Japan). Room conditions were standardised, with the temperature maintained at 23 ^∘^C, relative humidity of 50% and a 12-h/12-h light–dark cycle. Animals had free access to water and commercial food pellets. Mice used for experiments were killed by cervical dislocation.

### Fluorescence imaging for learning and evaluation

For the training dataset used for 11-fold cross-validation, 5522 time-series images of 11 early mouse embryos from the pronuclear stage to the maximum of the 53-cell stage were taken under a 3D confocal fluorescence microscope. The conditions of image acquisition are summarised in Supplementary Table 1. Each embryo had a different developmental rate and was at a different developmental stage (Supplementary Fig. 1). The test dataset consisted of 521 time-series images of four mouse embryos acquired under different imaging conditions (Supplementary Table 2).

### Immunostaining

Histone H2B-mCherry mRNA was injected into pronuclear stage embryos as described^2^. Embryos were fixed at room temperature in 4% paraformaldehyde, 0.1% polyvinyl alcohol in PBS for 30 min, permeabilised in 0.25% Triton-X 100 in PBS for 20 min and blocked in 3% bovine serum albumin in PBS for 1 h. Mouse monoclonal anti-Cdx2 (1:500, overnight, MU392-UC, BioGenex, San Ramon, CA) and rabbit polyclonal anti-Oct3/4 (1:500, sc-9081, Santa Cruz Biotechnology, Inc., Dallas, TX) were used as primary antibodies. Alexa Fluor-conjugated secondary antibodies (1:500; 1 h; Molecular Probes) were used. Laser scanning confocal images were acquired by using a CSU-W1 SoRa microscope (Yokogawa Electric Corp., Tokyo, JP).

### Ground truth creation

Using Fiji, an open-source platform for biological-image analysis,^65^ we manually created the ground truth for training dataset from fluorescence microscopic images at 11 time points in 11 mouse embryos (Supplementary Fig. 1). We excluded the nuclei of polar bodies from the ground truth. The ground truth to learn the task of nuclear identification was a spherical region with a diameter of 5 voxels; this region was based on the nuclear centre of gravity coordinates. This size is the maximum diameter at which adjacent nuclear centre regions do not contact each other. We also performed these tasks on the test dataset of mouse embryos as well as the datasets of *C. elegans* and *D. melanogaster* embryos.

### QCANet overview

QCANet consists of NSN, which learns the nuclear segmentation task, and NDN, which learns the nuclear identification task (Fig. 2); both NSN and NDN learn their tasks from the created ground truth. QCANet performs instance segmentation of the time-series 3D fluorescence microscopic images at each time point. The quantitative criteria of mouse development can be extracted from the acquired time-series instance segmentation image.

We implemented QCANet in Python 2.7 and used Chainer^66^, an open-source deep learning framework. We used NVIDIA Tesla K40 (operating frequency, 745 MHz; single precision floating point performance, 4.29 TFLOPS) and NVIDIA Tesla P100 (1189 MHz, 9.3 TFLOPS) for calculation of learning and segmentation. P100 is on Reedbush-H, a calculation server of the University of Tokyo Information Infrastructure Center.

### Pre-processing in QCANet

The objective of normalisation was to prevent the divergence of values and gradient disappearance in learning. The value of each voxel to be normalised ($I^{\prime}$) was defined by

1 $$I^{\prime} =\frac{I-{I}_{\min }}{{I}_{\max }-{I}_{\min }},$$

where *I* is the value of each voxel to be normalised, ${I}_{\max }$ is the maximum voxel value in the image and ${I}_{\min }$ is the minimum voxel value in the image. The value of $I^{\prime}$ was obtained for all the voxels in the image, and the range of the voxel values was [0, 1].

To fit the patch area within an image even if the voxel of interest was out of the image, mirror padding was performed by acquiring voxel values inside of *m* pixels from the edge of the image and extrapolating this mirror image to the outer edge. The patch size of QCANet was 128 voxels, so the size of the mirror-padded region was 64 voxels.

Because *x*, *y* and *z*-axis resolution in the microscopic image to be analysed was 0.8:0.8:1.75 μm (Supplementary Table 1), it was necessary to change it to the actual scale ratio of 1:1:1. Using bicubic interpolation, we interpolated 2.1875 times in the *z*-axis direction.

Because the number of samples of the ground truth was small, we performed data augmentation and increased the number of data four times for each training image by flipping on the *x*-axis, *y*-axis and both axes. Because the luminance bias in the *z*-axis direction (a feature of time-series 3D fluorescence microscopic images) is always constant, we did not expand the data in this direction.

### Nuclear segmentation network

We used Stochastic Gradient Descent (SGD) as an optimisation method for learning NSN. The structure of the network is based on 3D U-Net^23^, and parameter tuning suitable for the dataset was performed by Bayesian optimisation in SigOpt (https://sigopt.com). SigOpt was used as an optimisation platform. NSN had 1,146,896 parameters fewer than 3D U-Net (Supplementary Table 6).

The output function of NSN, called softmax, is defined by

2 $${y}_{k}=\frac{\exp ({x}_{k})}{{\Sigma }_{j = 1}^{K}\exp ({x}_{j})},$$

where *K* denotes the number of classes (nucleus or background region), *x* denotes each input from the final layer and *y* denotes the output value. The objective function of NSN, dice loss function^67^, is defined by

3 $$E=\frac{2\mathop{\sum }\nolimits_{i}^{N}{y}_{i}{g}_{i}}{\mathop{\sum }\nolimits_{i}^{N}{y}_{i}^{2}+\mathop{\sum }\nolimits_{i}^{N}{g}_{i}^{2}},$$

where *g* denotes the ground truth and *N* denotes the number of learning data. In the segmentation task, it is often a problem that labels (the number of pixels or voxels in the background and objects) are not balanced; the use of dice loss function as an objective function can suppress the influence of dataset label imbalance^67^.

### Nuclear Detection Network

We used Adam^68^ as an optimisation method for learning. The structure of the network was based on 3D U-Net^23^, and parameter tuning suitable for the dataset was performed by Bayesian optimisation in SigOpt. NDN had 44,447,940 parameters more than 3D U-Net (Supplementary Table 7). As in NSN, softmax and dice loss functions were used as the output and objective functions, respectively.

### Post-processing in QCANet

We performed (a) reinterpolation and (b) marker-based watershed transformation on the semantic segmentation image output from NSN and NDN. Reinterpolation restores the resolution of the image interpolated for segmentation and identification. Marker-based watershed divides the semantic segmentation region by watershed with the centre region of the identified nucleus as a marker. Post-processing enables QCANet to execute instance segmentation.

### Evaluation metrics for segmentation

An answer was considered correct when a voxel of an object region was classified as such (true positive, TP) or a voxel of a background region was classified as such (true negative, TN). An answer was considered incorrect when a voxel of a background region was classified as an object region (false positive, FP) or a voxel of an object region was classified as a background region (false negative, FN). Accordingly, IoU was defined as

4 $${\rm{IoU}}=\frac{{\rm{TP}}}{{\rm{TP}}+{\rm{FP}}+{\rm{FN}}},$$

where TP, FP and FN denote the numbers of voxels defined as above.

SEG was defined as

5 $${\rm{SEG}}=\mathop{\sum }\limits_{j}^{{N}_{i}}\frac{1}{{N}_{i}}\mathop{\max }\limits_{i}{\rm{IoU}}({y}_{i},{y}_{j}^{* }),$$

where *N*_*i*_ is the number of segmented nuclei, *y* is the segmented nuclear region, y* is the ground truth of the nuclear region, *i* is a label attached to the segmented nuclear region (*i* = 1, …, *N*_*i*_) and *j* is a label attached to the ground truth of the nuclear region. According to a previous study^40^, IoU was calculated only when (*y* ∩ y*) > 0.5 ⋅ y* as a constraint condition.

MUCov was defined as

6 $${\rm{MUCov}}=\mathop{\sum }\limits_{i}^{{N}_{j}}\frac{1}{{N}_{j}}\mathop{\max }\limits_{j}{\rm{IoU}}({y}_{i},{y}_{j}^{* }),$$

where *N*_*j*_ is the number of ground truth objects and other variables are as for SEG. The constraint condition was defined as (*y* ∩ y*) > 0.5 ⋅ y*.

### Model architecture and learning conditions of NSN

NSN hyperparameters were determined by Bayesian optimisation, and the model architecture of NSN was based on these hyperparameters (Supplementary Table 6). Epoch was fixed at 150 for learning. Using SGD and Adam, we evaluated learning the model. Because NSN trained by SGD performed nuclear segmentation with high accuracy, we adopted SGD- trained NSN for QCANet.

### Model architecture and learning conditions of NDN

NDN hyperparameters were determined by Bayesian optimisation, and the model architecture of NDN was based on these hyperparameters (Supplementary Table 7). Epoch was fixed at 150 for learning. Using SGD and Adam, we evaluated learning the model by NDN. Because NDN trained by Adam performed nuclear identification with high accuracy, we adopted Adam-trained NDN for QCANet.

### Training of previous algorithms

3D U-Net was trained by using reported hyperparameters^23^. The source code used in a previous study^39^ was used to implement 3D Mask R-CNN. Recommended values of hyperparameters were applied to 3D Mask R-CNN, but the number of candidates output by the Region Proposal Network was set to 200 as a result of tuning. Epoch was set at 150 for 3D U-Net and at 100 for 3D Mask R-CNN; at these values, the learning was judged to be sufficiently converged. Adam was used as an optimisation technique for both algorithms.

### Extraction of quantitative criteria from segmentation images

Nuclear number was extracted by counting the number of labels in segmentation images. Nuclear volume was extracted by converting the voxel number of the segmented nuclear region for each label to the actual scale. Nuclear surface area was extracted by converting the voxel number of the nuclear region that was in contact with the background region to the actual scale. The nuclear centre of gravity coordinates was calculated as the centre of gravity of the segmented nuclear region for each label. The synchrony of cell division was extracted from the time-series data for the nuclear number. The embryo was considered to have reached a certain stage if this stage lasted for at least 1 h.

### Classification of the nuclei of differentiated cells

The centre of gravity coordinates of the embryo were defined by using all the extracted nuclear centre of gravity coordinates at a particular time point. The distance from the centre of gravity coordinates of the embryo to those of the farthest cell nucleus was calculated as the radius *R* of the embryo. Then, for the radius *R*, we introduced the parameter *r* (0 ≤ *r* ≤ 1) as a threshold to classify inner cells and outer cells. Cells with the nuclei within *rR* were classified as inner cells and those with the nuclei outside *rR* as outer cells (Supplementary Fig. 7). Since the classification result at *r* = 0.4 was qualitatively in best agreement with the result obtained with specific markers (Supplementary Fig. 7b), *r* = 0.4 was adopted for the classification of inner vs. outer cells.

### Reporting summary

Further information on research design is available in the Nature Research Reporting Summary linked to this article.

## Supplementary information

Supplemental Material

Reporting Summary

Supplementary Video 1

## Data Availability

Part of training and testing datasets for mouse embryo 2 have been deposited to the Broad Bioimage Benchmark Collection (accession number BBBC050, see https://bbbc.broadinstitute.org/BBBC050). Data for *C*.elegans and *D.melanogaster* embryos were taken from the Cell Tracking Challenge (“*C.elegans* developing embryo” and “Developing *Drosophila Melanogaster* embryo”, see http://celltrackingchallenge.net/3d-datasets/).

## Code availability

The source code of QCANet is available from https://github.com/funalab/QCANet.
