# Supplementary material for: 3D convolutional neural networks-based segmentation to acquire quantitative criteria of the nucleus during mouse embryogenesis
Source: NPJ Syst Biol Appl. 2020 Oct 20;6:32. doi: 10.1038/s41540-020-00152-8 (PMC7575569; doi:10.1038/s41540-020-00152-8)
Supplement: Supplementary file 1 — Supplemental Material [file 41540_2020_152_MOESM1_ESM.pdf]

3D convolutional neural networks-based segmentation to acquire quantitative criteria of the nucleus during mouse embryogenesis

Yuta Tokuoka[1], Takahiro G Yamada[1], Daisuke Mashiko[2], Zenki Ikeda[2], Noriko F Hiroi[3], Tetsuya J Kobayashi[4], Kazuo Yamagata[2], and Akira Funahashi[1,*]

[1]Department of Biosciences and Informatics, Keio University, Kanagawa 223-8522, Japan
[2]Faculty of Biology-Oriented Science and Technology, Kindai University, Wakayama 649-6493, Japan
[3]Faculty of Pharmaceutical Sciences, Sanyo-Onoda City University, Yamaguchi 756-0884, Japan
[4]Institute of Industrial Science, The University of Tokyo, Tokyo 153-8505, Japan
[*]funa@bio.keio.ac.jp

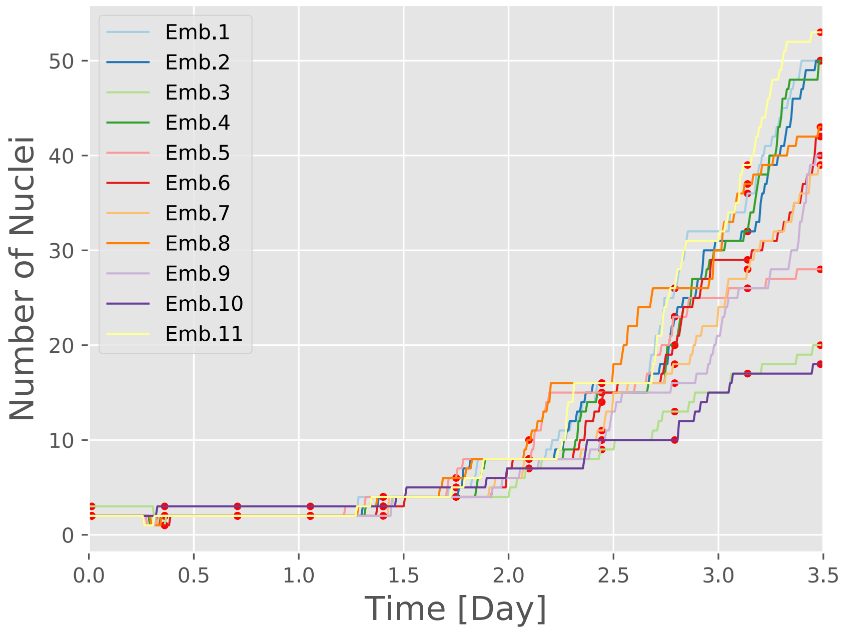

Supplementary Figure 1: A time-series plot of the ground truth created by using the training mouse embryo. Lines represent the number of nuclei per embryo; red circles represent time points used to create the ground truth.

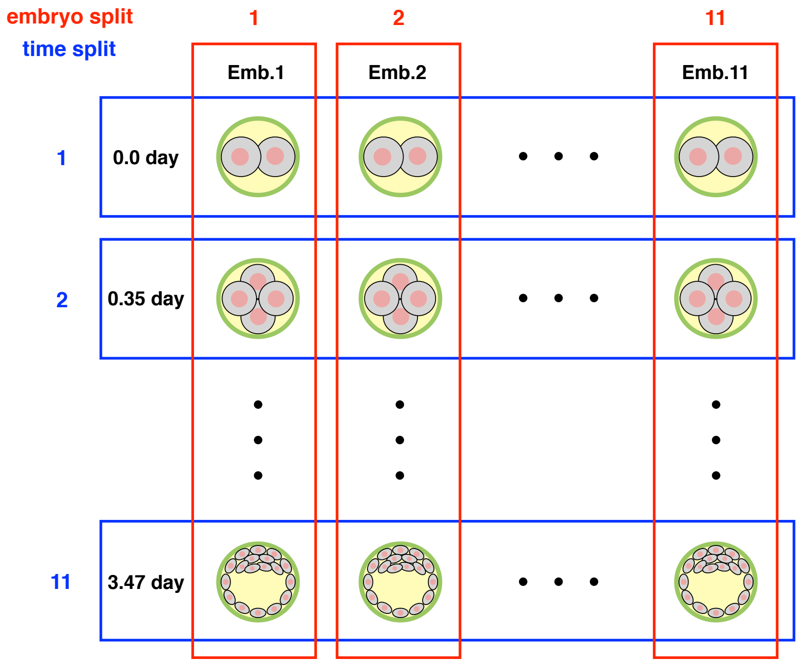

Supplementary Figure 2: Split datasets for cross-validation: a conceptual diagram.

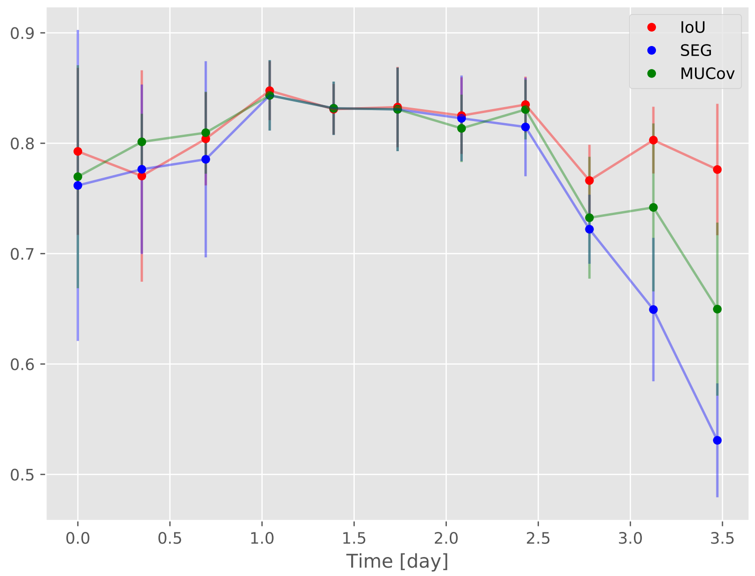

Supplementary Figure 3: Evaluation of cross-validation with the time-split dataset. Error bars represent standard deviation.

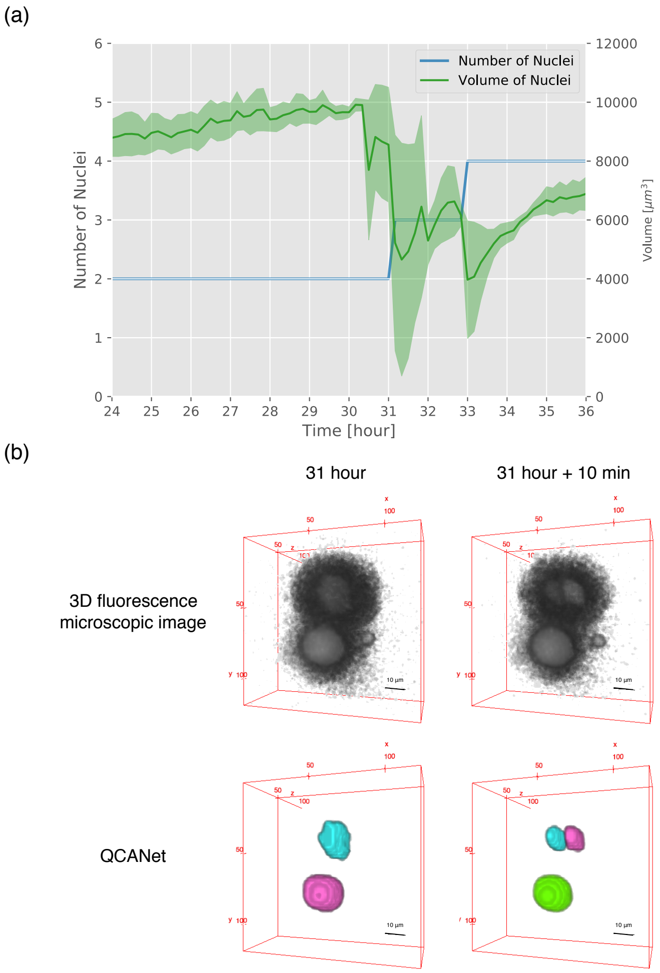

Supplementary Figure 4: (a) Time-series data of the nuclear number and nuclear volume in embryo 2 extracted by QCANet. For the nuclear volume, the mean and standard deviation are shown. (b) 3D fluorescence microscopic images and its instance segmentation results by QCANet.

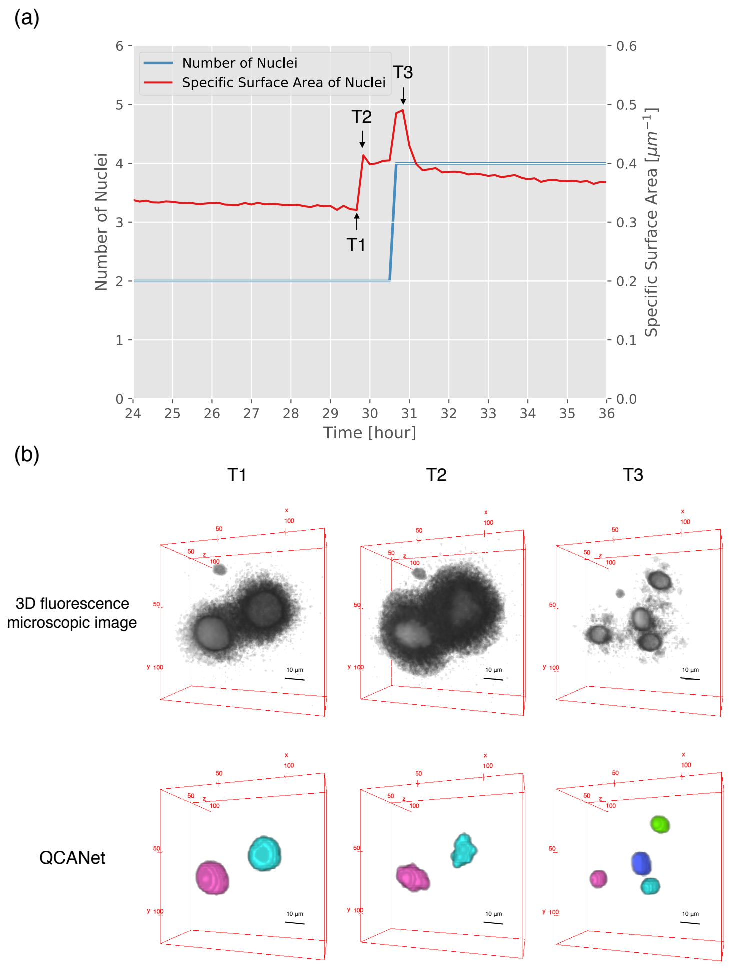

Supplementary Figure 5: (a) Time-series data of the nuclear number and nuclear specific surface area in embryo 1 extracted by QCANet. (b) 3D fluorescence microscopic images and its instance segmentation results by QCANet. The nuclear shape changed rapidly from T1 to T2. In T3 as after division, the specific surface area was maximised because the nuclear volume had decreased sharply.

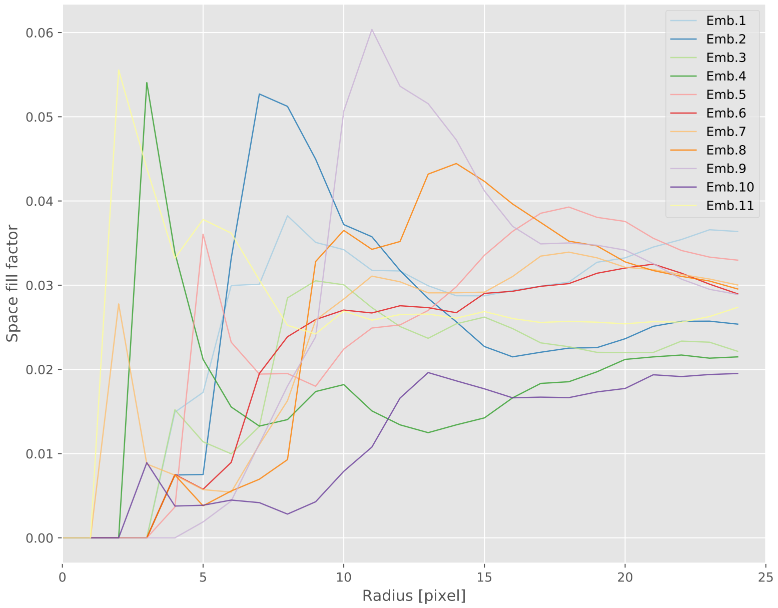

Supplementary Figure 6: The space fill factors from all-time data of the nuclear centre of gravity coordinates in each embryo. The ratio of the number of barycentric coordinates contained in the sphere of radius from the barycenter of the embryo is plotted.

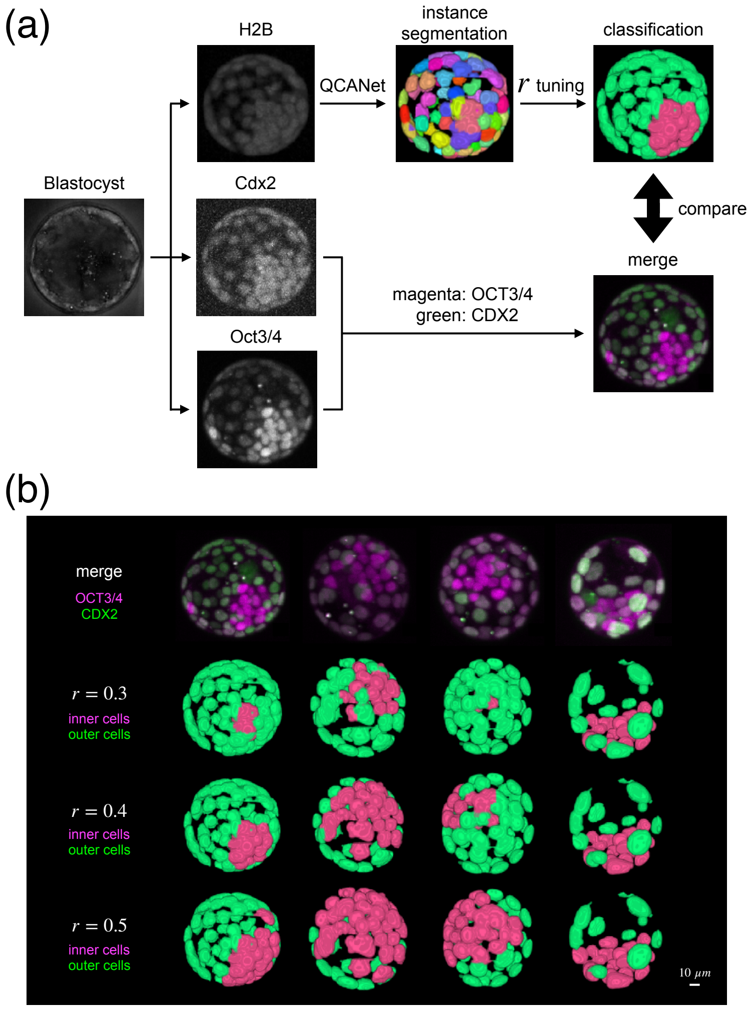

Supplementary Figure 7: Method for classification of inner and outer cells and tuning the results. (a) Conceptual diagram of the method to tune parameter r. (b) Result of r tuning. At $r = 0.4$, the merge and classification results are qualitatively the best.

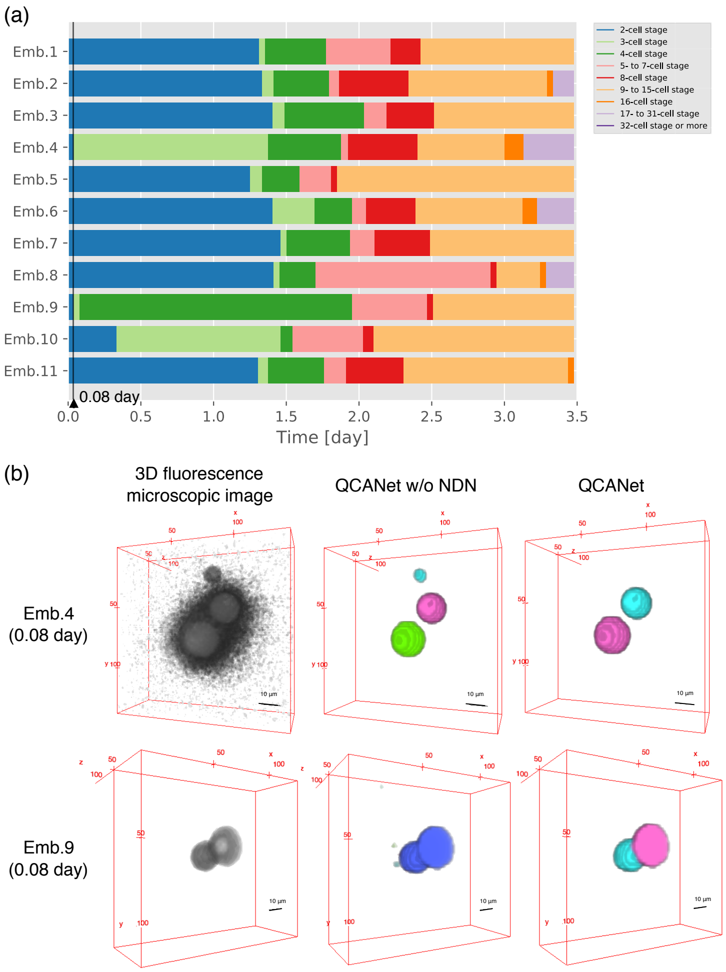

Supplementary Figure 8: Comparison of QCANet and QCANet w/o NDN in acquisition of the synchrony of cell division. (a) Acquisition of the synchrony of cell division by QCANet w/o NDN. (b) An example of the cause of failing to acquire the synchrony of cell division.

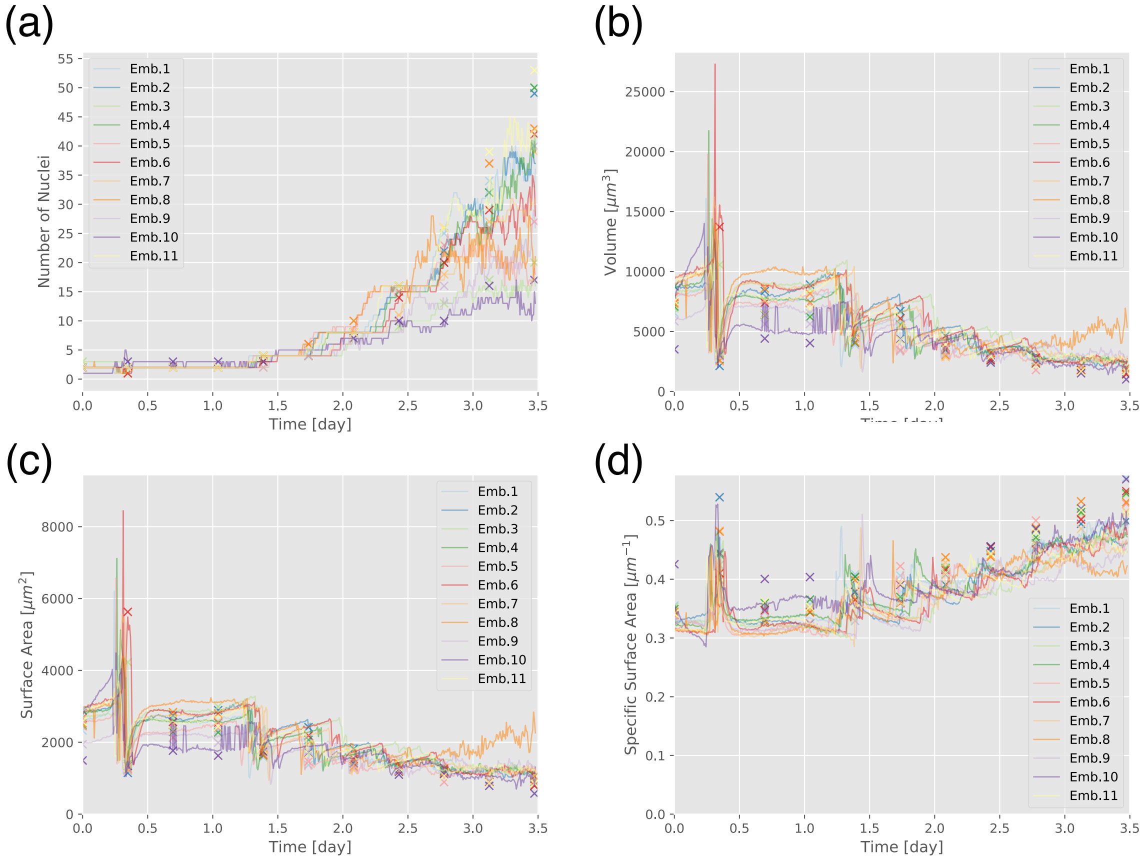

Supplementary Figure 9: Comparison of quantitative criteria extracted by QCANet and ground truth. Each line is plotted in the same way as in Figure 5. The cross marks represent the correct answers created from ground truth. (a) Nuclear number. (b) Nuclear volume. (c) Nuclear surface area. (d) Nuclear specific surface area.

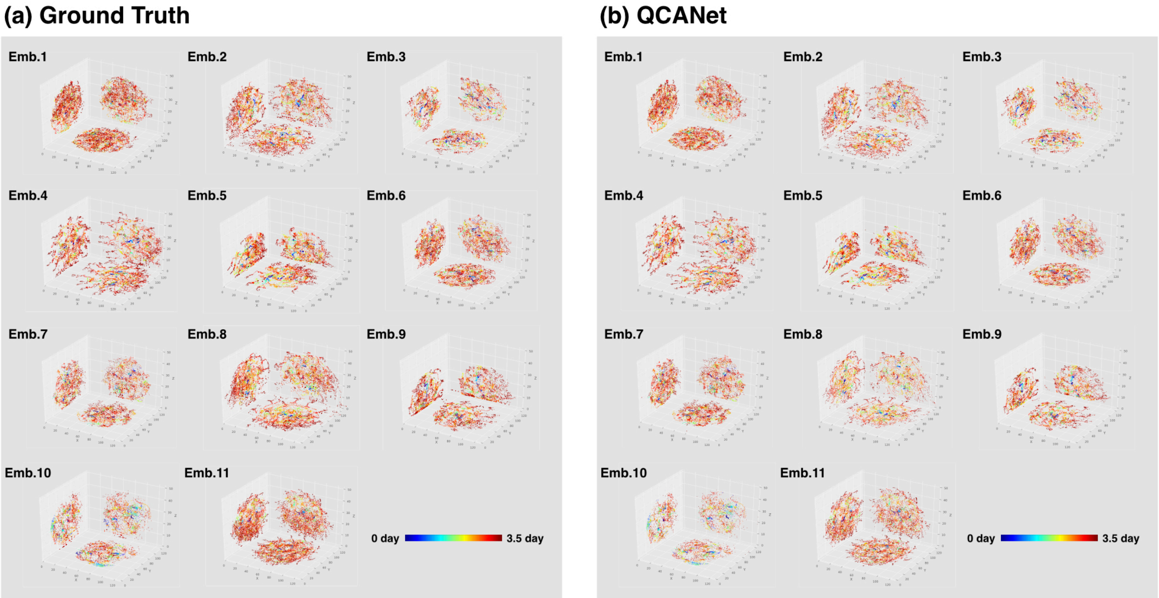

Supplementary Figure 10: Comparison of the time-series data of the nuclear centre of gravity coordinates extracted by ground truth and QCANet. Colour shift from cold to warm indicates the course of development. In each panel, the results are displayed in 2D (XY, XZ, and YZ cross-sections).

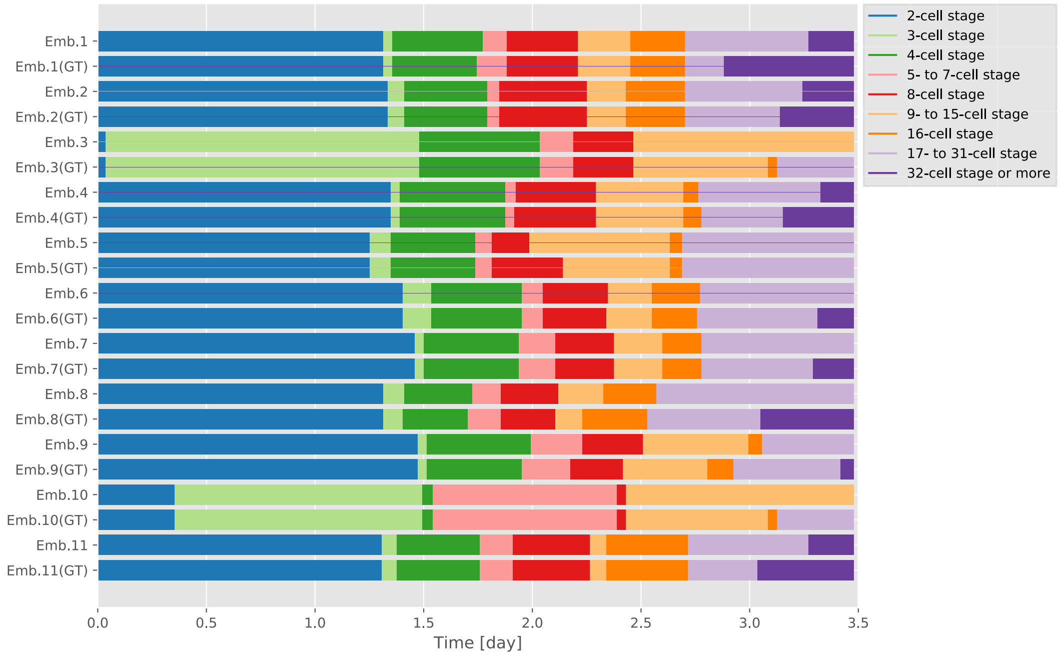

Supplementary Figure 11: Comparison of the synchrony of cell division extracted by ground truth and QCANet. GT, ground truth.

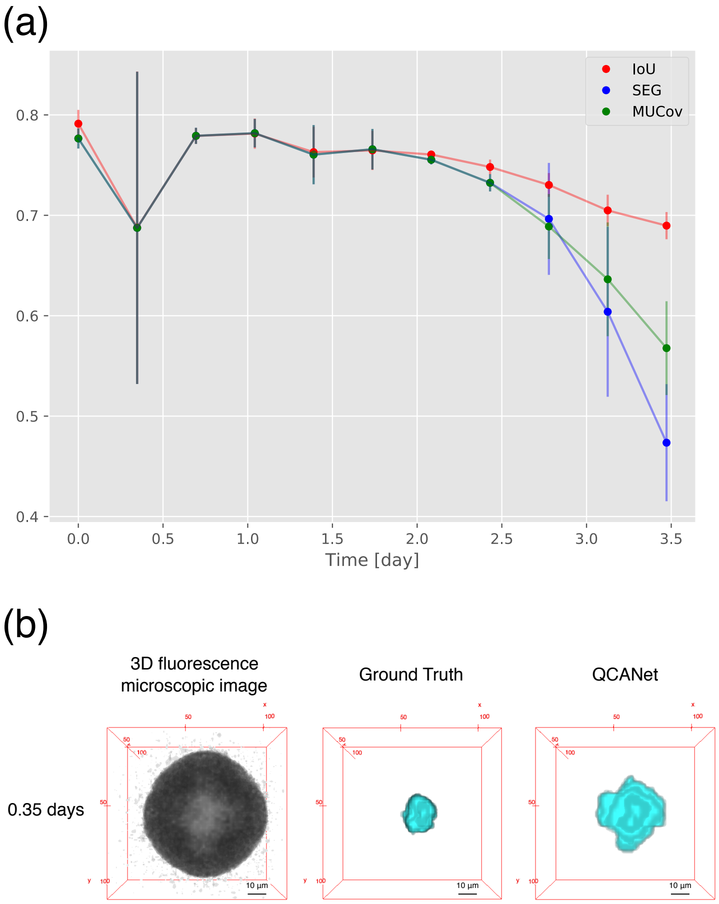

Supplementary Figure 12: Evaluation of the test dataset of mouse embryos at each time point. (a) IoU, SEG, and MUCov values. Error bars represent standard deviation. (b) Segmentation by QCANet at 0.35 days.

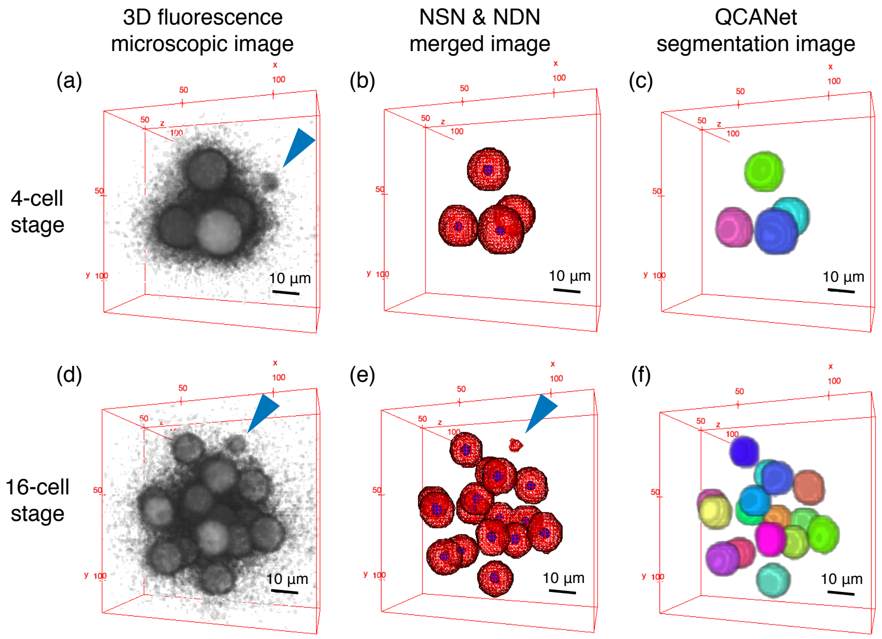

Supplementary Figure 13: Recognition of polar bodies and their exclusion from segmentation targets. (a-c) Both NSN and NDN excluded the polar body (blue arrowhead). (d-f) NSN performed segmentation of the polar body, but NDN excluded it. (a,d) The acquired 3D images. (b,e) Red mesh, nuclear segmentation by NSN; blue mesh, nuclear identification by NDN. (c,f) Instance segmentation by QCANet excluded polar bodies in both cases.

Supplementary Table 1: Acquisition conditions of time-series 3D fluorescence microscopic images of mouse embryos for the training dataset.

Observation target	Mouse embryo
Fluorescent protein	H2B-mRFP1
Delivery of fluorescent protein	mRNA Microinjection
Microscope	IX71 (Olympus, Tokyo, Japan)
Confocal system	CSU-X1 (YOKOGAWA, Tokyo, Japan)
Image size [$voxel$]	512×512×51
Spatial resolution ($x : y : z$) [$\mu m/voxel$]	0.8 : 0.8 : 1.75
Time resolution [min]	10
Number of time slices	502
Number of embryo analyzed	11

Supplementary Table 2: Acquisition conditions of time-series 3D fluorescence microscopic images of mouse embryos for the test dataset.

Observation target	Mouse embryo
Fluorescent protein	H2B-mCherry
Delivery of fluorescent protein	mRNA Microinjection
Microscope and Confocal system	CV1000 (Yokogawa Electric Corp, Tokyo, Japan)
Image size [$voxel$]	512×512×51
Spatial resolution ($x : y : z$) [$\mu m/voxel$]	0.8 : 0.8 : 2.0
Time resolution [min]	10
Number of time slices	502
Number of embryo analyzed	4

Supplementary Table 3: Quantitative evaluation of segmentation accuracy for the mouse dataset. For the evaluation of training, 11-fold cross-validation was performed. Values in parentheses are standard deviation (Std).

Metrics	Algorithm	Emb.1	Emb.2	Emb.3	Emb.4	Emb.5	Emb.6	Emb.7	Emb.8	Emb.9	Emb.10	Emb.11	Mean (Std)
IoU	3D U-Net	0.692 (0.070)	0.761 (0.115)	0.708 (0.078)	0.649 (0.050)	0.700 (0.031)	0.695 (0.068)	0.677 (0.056)	0.652 (0.058)	0.698 (0.041)	0.635 (0.124)	0.696 (0.058)	0.688 (0.033)
	3D Mask R-CNN	0.595 (0.257)	0.566 (0.217)	0.728 (0.166)	0.670 (0.228)	0.589 (0.227)	0.665 (0.208)	0.677 (0.219)	0.568 (0.234)	0.531 (0.229)	0.744 (0.127)	0.642 (0.310)	0.634 (0.066)
	QCANet w/o NDN	0.817 (0.031)	**0.836 (0.082)**	**0.790 (0.049)**	0.806 (0.036)	**0.856 (0.027)**	0.813 (0.036)	**0.821 (0.025)**	0.776 (0.052)	**0.839 (0.030)**	**0.786 (0.048)**	**0.804 (0.016)**	0.813 (0.023)
	QCANet	**0.817 (0.030)**	**0.836 (0.082)**	0.789 (0.049)	**0.807 (0.036)**	0.856 (0.028)	**0.814 (0.035)**	0.821 (0.026)	**0.781 (0.045)**	0.835 (0.034)	0.785 (0.050)	0.803 (0.016)	**0.813 (0.022)**
SEG	3D U-Net	0.467 (0.285)	0.548 (0.263)	0.597 (0.193)	0.484 (0.208)	0.500 (0.241)	0.536 (0.260)	0.487 (0.264)	0.399 (0.243)	0.375 (0.193)	0.501 (0.199)	0.526 (0.278)	0.493 (0.061)
	3D Mask R-CNN	0.540 (0.297)	0.541 (0.247)	0.677 (0.220)	0.624 (0.252)	0.532 (0.241)	0.632 (0.253)	0.649 (0.254)	0.494 (0.261)	0.473 (0.229)	0.625 (0.187)	0.624 (0.329)	0.583 (0.065)
	QCANet w/o NDN	0.624 (0.275)	0.639 (0.233)	0.693 (0.175)	0.648 (0.195)	0.631 (0.251)	0.667 (0.263)	0.616 (0.270)	0.554 (0.297)	0.616 (0.227)	0.644 (0.165)	0.650 (0.230)	0.635 (0.033)
	QCANet	**0.772 (0.114)**	**0.784 (0.138)**	**0.760 (0.103)**	**0.776 (0.090)**	**0.805 (0.128)**	**0.781 (0.097)**	**0.784 (0.089)**	**0.692 (0.179)**	**0.755 (0.125)**	**0.701 (0.150)**	**0.762 (0.097)**	**0.761 (0.033)**
MUCov	3D U-Net	0.486 (0.252)	0.438 (0.216)	0.516 (0.223)	0.483 (0.148)	0.455 (0.147)	0.495 (0.232)	0.405 (0.224)	0.278 (0.119)	0.278 (0.208)	0.249 (0.125)	0.484 (0.194)	0.415 (0.095)
	3D Mask R-CNN	0.707 (0.185)	0.750 (0.092)	0.766 (0.103)	**0.804 (0.066)**	0.781 (0.089)	0.759 (0.107)	0.771 (0.101)	0.675 (0.168)	0.705 (0.135)	**0.782 (0.074)**	0.768 (0.170)	0.752 (0.037)
	QCANet w/o NDN	0.721 (0.148)	0.707 (0.180)	0.742 (0.107)	0.708 (0.112)	0.730 (0.116)	0.721 (0.174)	0.703 (0.167)	0.671 (0.173)	0.671 (0.198)	0.623 (0.132)	0.747 (0.093)	0.704 (0.035)
	QCANet	**0.794 (0.062)**	**0.811 (0.106)**	**0.772 (0.081)**	0.797 (0.051)	**0.825 (0.065)**	**0.800 (0.061)**	**0.801 (0.053)**	**0.735 (0.109)**	**0.818 (0.067)**	0.758 (0.101)	**0.782 (0.058)**	**0.790 (0.026)**

Supplementary Table 4: Quantitative evaluation of segmentation accuracy for the *Caenorhabditis elegans* dataset. For the evaluation of training, 3-fold cross-validation was performed. Values in parentheses are standard deviation (Std).

Metrics	Algorithm	Emb.1	Emb.2	Emb.3	Mean (Std)
IoU	3D U-Net	0.430 (0.032)	**0.673 (0.103)**	**0.530 (0.065)**	**0.544 (0.100)**
	3D Mask R-CNN	**0.534 (0.107)**	0.333 (0.088)	0.450 (0.080)	0.439 (0.082)
	QCANet w/o NDN	0.353 (0.047)	0.621 (0.018)	0.479 (0.036)	0.484 (0.109)
	QCANet	0.356 (0.049)	0.628 (0.021)	0.479 (0.039)	0.488 (0.111)
SEG	3D U-Net	0.235 (0.184)	0.373 (0.273)	0.313 (0.177)	0.307 (0.056)
	3D Mask R-CNN	**0.376 (0.196)**	0.100 (0.111)	0.335 (0.160)	0.270 (0.122)
	QCANet w/o NDN	0.206 (0.168)	0.347 (0.231)	0.298 (0.171)	0.284 (0.058)
	QCANet	0.277 (0.111)	**0.456 (0.106)**	**0.351 (0.087)**	**0.361 (0.073)**
MUCov	3D U-Net	0.002 (0.001)	0.003 (0.002)	0.003 (0.002)	0.003 (0.000)
	3D Mask R-CNN	**0.411 (0.087)**	0.077 (0.084)	**0.370 (0.088)**	0.286 (0.149)
	QCANet w/o NDN	0.023 (0.012)	0.145 (0.081)	0.075 (0.043)	0.081 (0.050)
	QCANet	0.265 (0.089)	**0.377 (0.153)**	0.357 (0.080)	**0.333 (0.049)**

Supplementary Table 5: Quantitative evaluation of segmentation accuracy for the *Drosophila melanogaster* dataset. For the evaluation of training, 3-fold cross-validation was performed. Values in parentheses are standard deviation (Std).

Metrics	Algorithm	Emb.1	Emb.2	Emb.3	Mean (Std)
IoU	3D U-Net	**0.733 (0.015)**	**0.652 (0.006)**	0.608 (0.014)	**0.664 (0.052)**
	3D Mask R-CNN	0.368 (0.001)	0.348 (0.003)	0.381 (0.001)	0.366 (0.014)
	QCANet w/o NDN	0.601 (0.020)	0.625 (0.002)	0.635 (0.006)	0.620 (0.014)
	QCANet	0.602 (0.020)	0.627 (0.002)	**0.637 (0.006)**	0.622 (0.015)
SEG	3D U-Net	0.004 (0.000)	0.000 (0.000)	0.000 (0.000)	0.001 (0.002)
	3D Mask R-CNN	0.096 (0.006)	0.103 (0.006)	0.105 (0.003)	0.101 (0.004)
	QCANet w/o NDN	0.001 (0.000)	0.009 (0.002)	0.005 (0.000)	0.005 (0.003)
	QCANet	**0.345 (0.006)**	**0.348 (0.014)**	**0.315 (0.005)**	**0.336 (0.015)**
MUCov	3D U-Net	0.004 (0.000)	0.005 (0.001)	0.001 (0.001)	0.003 (0.002)
	3D Mask R-CNN	0.259 (0.010)	0.236 (0.002)	0.241 (0.005)	0.245 (0.010)
	QCANet w/o NDN	0.006 (0.006)	0.072 (0.008)	0.045 (0.001)	0.041 (0.027)
	QCANet	**0.349 (0.005)**	**0.334 (0.005)**	**0.338 (0.010)**	**0.340 (0.006)**

Supplementary Table 6: The model architecture of NSN. CBR, Convolution + Batch Normalization + ReLU. k, kernel size. s, stride. p, pad size. f, filter.

Layer	Hyperparameter	Description
1	3D CBR	k = 3, s = 1, p = 1, f = 16
2	3D CBR	k = 3, s = 1, p = 1, f = 32
3	3D Max Pooling	k = 2, s = 2
4	3D CBR	k = 3, s = 1, p = 1, f = 32
5	3D CBR	k = 3, s = 1, p = 1, f = 64
6	3D Max Pooling	k = 2, s = 2
7	3D CBR	k = 3, s = 1, p = 1, f = 64
8	3D CBR	k = 3, s = 1, p = 1, f = 128
9	3D Deconvolution	k = 2, s = 2, p = 0, f = 128
10	Concatenation	Layer 5 + 9
11	3D CBR	k = 3, s = 1, p = 1, f = 64
12	3D CBR	k = 3, s = 1, p = 1, f = 64
13	3D Deconvolution	k = 2, s = 2, p = 0, f = 64
14	Concatenation	Layer 2 + 13
15	3D CBR	k = 3, s = 1, p = 1, f = 32
16	3D CBR	k = 3, s = 1, p = 1, f = 32
17	3D Convolution	k = 1, s = 1, p = 0, f = 2

Supplementary Table 7: The model architecture of NDN. CBR, Convolution + Batch Normalization + ReLU. k, kernel size. s, stride. p, pad size. f, filter.

Layer	Hyperparameter	Description
1	3D CBR	k = 5, s = 1, p = 1, f = 12
2	3D CBR	k = 5, s = 1, p = 1, f = 24
3	3D Max Pooling	k = 2, s = 2
4	3D CBR	k = 5, s = 1, p = 1, f = 24
5	3D CBR	k = 5, s = 1, p = 1, f = 48
6	3D Max Pooling	k = 2, s = 2
7	3D CBR	k = 5, s = 1, p = 1, f = 48
8	3D CBR	k = 5, s = 1, p = 1, f = 96
9	3D Max Pooling	k = 2, s = 2
10	3D CBR	k = 5, s = 1, p = 1, f = 96
11	3D CBR	k = 5, s = 1, p = 1, f = 192
12	3D Max Pooling	k = 2, s = 2
13	3D CBR	k = 5, s = 1, p = 1, f = 192
14	3D CBR	k = 5, s = 1, p = 1, f = 384
15	3D Deconvolution	k = 2, s = 2, p = 0, f = 384
16	Concatenation	Layer 11 + 15
17	3D CBR	k = 5, s = 1, p = 1, f = 192
18	3D CBR	k = 5, s = 1, p = 1, f = 192
19	3D Deconvolution	k = 2, s = 2, p = 0, f = 192
20	Concatenation	Layer 8 + 19
21	3D CBR	k = 5, s = 1, p = 1, f = 96
22	3D CBR	k = 5, s = 1, p = 1, f = 96
23	3D Deconvolution	k = 2, s = 2, p = 0, f = 96
24	Concatenation	Layer 5 + 23
25	3D CBR	k = 5, s = 1, p = 1, f = 48
26	3D CBR	k = 5, s = 1, p = 1, f = 48
27	3D Deconvolution	k = 2, s = 2, p = 0, f = 48
28	Concatenation	Layer 2 + 27
29	3D CBR	k = 5, s = 1, p = 1, f = 24
30	3D CBR	k = 5, s = 1, p = 1, f = 24
31	3D Convolution	k = 1, s = 1, p = 0, f = 2

Supplementary Video 1: Instance segmentation of the time-series 3D fluorescence microscopic images of early mouse embryos. The images of individual embryos are shown.
