# Supplementary material for: 3D convolutional neural networks-based segmentation to acquire quantitative criteria of the nucleus during mouse embryogenesis
Source: NPJ Syst Biol Appl. 2020 Oct 20;6:32. doi: 10.1038/s41540-020-00152-8 (PMC7575569; doi:10.1038/s41540-020-00152-8)
Supplement: Supplementary file 2 — Reporting Summary [file 41540_2020_152_MOESM2_ESM.pdf]

nature research

Corresponding author(s): Akira Funahashi

Last updated by author(s): Aug 29, 2020

Reporting Summary

Nature Research wishes to improve the reproducibility of the work that we publish. This form provides structure for consistency and transparency in reporting. For further information on Nature Research policies, see our Editorial Policies and the Editorial Policy Checklist.

Statistics

For all statistical analyses, confirm that the following items are present in the figure legend, table legend, main text, or Methods section.

n/a	Confirmed	
☐	☒	The exact sample size (*n*) for each experimental group/condition, given as a discrete number and unit of measurement
☐	☒	A statement on whether measurements were taken from distinct samples or whether the same sample was measured repeatedly
☒	☐	The statistical test(s) used AND whether they are one- or two-sided<br>*Only common tests should be described solely by name; describe more complex techniques in the Methods section.*
☒	☐	A description of all covariates tested
☒	☐	A description of any assumptions or corrections, such as tests of normality and adjustment for multiple comparisons
☐	☒	A full description of the statistical parameters including central tendency (e.g. means) or other basic estimates (e.g. regression coefficient) AND variation (e.g. standard deviation) or associated estimates of uncertainty (e.g. confidence intervals)
☒	☐	For null hypothesis testing, the test statistic (e.g. *F*, *t*, *r*) with confidence intervals, effect sizes, degrees of freedom and *P* value noted<br>*Give P values as exact values whenever suitable.*
☒	☐	For Bayesian analysis, information on the choice of priors and Markov chain Monte Carlo settings
☒	☐	For hierarchical and complex designs, identification of the appropriate level for tests and full reporting of outcomes
☒	☐	Estimates of effect sizes (e.g. Cohen's *d*, Pearson's *r*), indicating how they were calculated

Our web collection on statistics for biologists contains articles on many of the points above.

Software and code

Policy information about availability of computer code

Data collection	The training dataset consisted of 5,522 time-series images of 11 mouse embryos and the test dataset consisted of 521 time-series images of four mouse embryos: MetaMorph software ver. 7.7.10 (Molecular Devices; Commercial software)<br>Immunostaining Images: Micro-Manager 2.0 (Open Source)
Data analysis	All the software code used in the analysis of this study is described in Methods.

For manuscripts utilizing custom algorithms or software that are central to the research but not yet described in published literature, software must be made available to editors and reviewers. We strongly encourage code deposition in a community repository (e.g. GitHub). See the Nature Research guidelines for submitting code & software for further information.

Data

Policy information about availability of data

All manuscripts must include a data availability statement. This statement should provide the following information, where applicable:

- Accession codes, unique identifiers, or web links for publicly available datasets
- A list of figures that have associated raw data
- A description of any restrictions on data availability

Part of training and testing datasets for mouse embryo 2 have been deposited to the Broad Bioimage Benchmark Collection (accession number BBBC050, see https://bbbc.broadinstitute.org/BBBC050). Data for C.elegans and D.melanogaster embryos were taken from the Cell Tracking Challenge ("C.elegans developing embryo" and "Developing Drosophila Melanogaster embryo", see http://celltrackingchallenge.net/3d-datasets/). The data used to generate Figures 3–8 and the movie are available from the authors on reasonable request (see author contributions for specific data sets).

Field-specific reporting

Please select the one below that is the best fit for your research. If you are not sure, read the appropriate sections before making your selection.

☒ Life sciences ☐ Behavioural & social sciences ☐ Ecological, evolutionary & environmental sciences

For a reference copy of the document with all sections, see nature.com/documents/nr-reporting-summary-flat.pdf

Life sciences study design

All studies must disclose on these points even when the disclosure is negative.

Sample size	For the training dataset, 5,522 time-series images of 11 early mouse embryos were taken under a 3D confocal fluorescence microscope. For the test dataset, 521 time-series images of four early mouse embryos were taken under a 3D confocal fluorescence microscope. The above can be found in the "Fluorescence Imaging for Learning and Evaluation" section.
Data exclusions	No data were excluded from the analyses.
Replication	Experiments were performed in eleven and four biological replicates for training and test dataset, respectively.
Randomization	This study did not require randomization processing because all 11 and four mouse embryos acquired under the same experimental conditions were subjected to the same image analysis.
Blinding	This study did not require blinding because all 11 and four mouse embryos acquired under the same experimental conditions were subjected to the same image analysis.

Reporting for specific materials, systems and methods

We require information from authors about some types of materials, experimental systems and methods used in many studies. Here, indicate whether each material, system or method listed is relevant to your study. If you are not sure if a list item applies to your research, read the appropriate section before selecting a response.

Materials & experimental systems

n/a	Involved in the study	
☐	☒	Antibodies
☒	☐	Eukaryotic cell lines
☒	☐	Palaeontology and archaeology
☐	☒	Animals and other organisms
☒	☐	Human research participants
☒	☐	Clinical data
☒	☐	Dual use research of concern

Methods

n/a	Involved in the study	
☒	☐	ChIP-seq
☒	☐	Flow cytometry
☒	☐	MRI-based neuroimaging

Antibodies

Antibodies used	Mouse monoclonal anti-Cdx2 (1:500, overnight, MU392-UC, BioGenex, San Ramon, CA) and rabbit polyclonal anti-Oct3/4 (1:500, sc-9081, Santa Cruz Biotechnology, Inc., Dallas, TX) were used as primary antibodies. Alexa Fluor–conjugated secondary antibodies (1:500; 1 h; Molecular Probes) were used.
Validation	anti-CDX2: http://store.biogenex.com/us/anti-cdx-2-clone-cdx2-226.html<br>anti-Oct3/4: http://datasheets.scbt.com/sc-9081.pdf

Animals and other organisms

Policy information about studies involving animals; ARRIVE guidelines recommended for reporting animal research

Laboratory animals	ICR strain multi clonal hybrid strain mice named Jcl: MCH (ICR) and slc: ICR, male and female, 10-13 weeks.
Wild animals	The study did not involve wild animals.
Field-collected samples	The study did not involve samples collected from the field.
Ethics oversight	Osaka University and Kindai University

Note that full information on the approval of the study protocol must also be provided in the manuscript.
